# Glycoprotein IIb/IIIa Inhibitors Use and Outcome after Percutaneous Coronary Intervention for Non-ST Elevation Myocardial Infarction

**DOI:** 10.1155/2014/643981

**Published:** 2014-05-08

**Authors:** J. P. Howard, D. A. Jones, S. Gallagher, K. Rathod, S. Antoniou, P. Wright, C. Knight, A. Mathur, R. Weerackody, A. Wragg

**Affiliations:** ^1^Department of Cardiology, Barts Health NHS Trust, London E2 9JX, UK; ^2^Department of Clinical Pharmacology, William Harvey Research Institute, Queen Mary University of London, London EC1M 6BQ, UK; ^3^NIHR Cardiovascular Biomedical Research Unit, London Chest Hospital, London E2 9JX, UK

## Abstract

*Aims*. We investigate the effect of glycoprotein IIb/IIIa (GP IIb/IIIa) inhibitors on long-term outcomes following percutaneous coronary intervention (PCI) after non-ST elevation myocardial infarction (NSTEMI). Meta-analyses indicate that these agents are associated with improved short-term outcomes. However, many trials were undertaken before the routine use of P2Y_12_ inhibitors. Recent studies yield conflicting results and registry data have suggested that GP IIb/IIIa inhibitors may cause more bleeding than what trials indicate. *Methods and Results*. This retrospective observational study involves 3047 patients receiving dual-antiplatelet therapy who underwent PCI for NSTEMI. Primary outcome was all-cause mortality. Major adverse cardiac events (MACE) were a secondary outcome. Mean follow-up was 4.6 years. Patients treated with GP IIb/IIIa inhibitors were younger with fewer comorbidities. Although the unadjusted Kaplan-Meier analysis suggested that GP IIb/IIIa inhibitor use was associated with improved outcomes, multivariate analysis (including propensity scoring) showed no benefit for either survival (*P* = 0.136) or MACE (*P* = 0.614). GP IIb/IIIa inhibitor use was associated with an increased risk of major bleeding (*P* = 0.021). *Conclusion*. Although GP IIb/IIIa inhibitor use appeared to improve outcomes after PCI for NSTEMI, patients who received GP IIb/IIIa inhibitors tended to be at lower risk. After multivariate adjustment we observed no improvement in MACE or survival and an increased risk of major bleeding.

## 1. Introduction

Percutaneous coronary intervention (PCI) has been shown to improve clinical outcomes in non-ST elevation myocardial infarction (NSTEMI) [1]. These patients typically receive multiple antiplatelet and anticoagulative therapies as per current guidelines [2, 3]. Glycoprotein IIb/IIIa receptor inhibitors are potent antiplatelet agents which have been shown in some trials to improve outcomes in patients undergoing PCI post-NSTEMI [4–8]. Meta-analyses involving 29,570 patients with NSTEMI/UA (non-ST elevation myocardial infarction or unstable angina) have shown GP IIb/IIIa inhibitors to be associated with a reduction in death or nonfatal myocardial infarction at 30 days [4]. Although positive studies have suggested a potential for a 30–40% reduction in the composite of death and MI, favouring the use of GP IIb/IIIa inhibitors [2], these trials were carried out before the routine use of clopidogrel or other P2Y_12_ inhibitors.

Studies performed with the use of clopidogrel have yielded conflicting results. The ISAR-REACT 2 trial [7] demonstrated reduced short-term mortality and cardiac events with abciximab. However, other studies such as ACUITY Timing [9] demonstrated no benefit from early GP IIb/IIIa inhibition to reduce the risk of major bleeds. Furthermore, registry data indicates that GP IIb/IIIa inhibitors are less safe than trial data would indicate and that their benefits may not extend to patient groups not recruited to clinical trials [10].

Current ESC guidelines state that “it is reasonable to combine a GP IIb/IIIa receptor inhibitor with aspirin and a P2Y_12_ inhibitor for patients with NSTE-ACS undergoing PCI with a high risk of procedural MI and without a high risk of bleeding” [3]. However, concerns over side effect profiles have led to their reduced usage in current practice, especially with the addition of newer more potent oral antiplatelet drugs.

We therefore investigated the outcome of patients treated by PCI for NSTEMI at our institution by comparing patients who received GP IIb/IIIa inhibitors to those who did not. We looked at long-term mortality, inhospital MACE, and major inhospitable bleeding complications.

## 2. Methods

We undertook an observational cohort study to investigate the relationship between the use of GP IIb/IIIa inhibitors and long-term outcomes after PCI for NSTEMI at a single high-volume cardiac centre in East London. The study period was from October 2003 to July 2011. During the study period 3321 patients underwent PCI for NSTEMI. Of these, 3089 (93.0%) had complete datasets and NHS numbers and were included in the analysis. NSTEMI was diagnosed in NSTE-ACS patients if the troponin T or I was above the reference range of the assay used. The group of patients excluded from the study due to incomplete data entry or absent NHS number contained a similar proportion of GP IIb/IIIa use. Patients with slow or no-reflow phenomenon after PCI (42 patients) were excluded as all these patients received GP IIb/IIIa inhibition. 3047 patients remained for analysis.

Standard PCI protocol included preloading with aspirin 300 mg and clopidogrel 600 mg. All patients were prescribed 75 mg aspirin and 75 mg clopidogrel maintenance therapy. Clopidogrel maintenance therapy was recommended for 1 year after intervention. Intravenous GP IIb/IIIa inhibitor was administered at the operator's discretion with the choice of agent based on the current local guidelines, which for the majority of patients was abciximab. Abciximab was administered as an intravenous bolus of 0.25 mg/kg before/during PCI followed by a continuous infusion of 0.125 mcg/kg/min (to a maximum of 10 *μ*g/min) for 12 hours. Eptifibatide was administered as an intravenous bolus of 180 mcg/kg before/at the start of PCI followed by a continuous infusion of 2 mcg/kg/min for 12 hours following the procedure. Tirofiban was administered as a loading dose of 400 ng/kg/min for 30 minutes followed by a continuous infusion of 150 ng/kg/minute for 18–24 hours following the procedure. Contraindications for GP IIb/IIIa inhibitor use included previous major gastrointestinal bleeding, previous haemorrhagic stroke, chronic renal impairment, thrombocytopenia, and concurrent oral anticoagulant use. GP IIb/IIIa inhibitor infusions were stopped if there was an adverse reaction, reduction in platelet count from baseline of >50%, or minor or major bleeding according to the TIMI bleeding criteria. All patients received unfractionated heparin at the time of PCI with an initial loading dose of 85 units per kilogram, with the dose adjusted to maintain an ACT > 300 s. All patients received preprocedural enoxaparin at 1 mg/kg subcutaneously twice daily for up to 5 days (750 mcg/kg twice daily in patients aged above 75). Bivalirudin was not available during the study period.

Data were prospectively entered into a clinical PCI database, based on the British Cardiovascular Intervention Society (BCIS) dataset [11], at the time of the procedure. Data collected included patient characteristics (age, prior myocardial infarction (MI), percutaneous coronary intervention (PCI), coronary artery bypass grafting (CABG), hypertension, diabetes mellitus, hypercholesterolemia, peripheral vascular disease (PVD), NYHA class, smoking status, chronic renal impairment (CRF: creatinine > 200 *μ*mol/L or on renal replacement therapy), LV function and cardiogenic shock), and procedure-related data (indications for PCI, target vessel, number of diseased vessels, use of IVUS/pressure wire, and use of drug-eluting stent and GP IIb/IIIa inhibitor).

Procedural complications and major adverse cardiac events (MACE) were recorded prospectively. MACE were defined as death, myocardial infarction (new pathologic Q waves in the distribution of the treated coronary artery with an increase of creatine kinase-MB to 2 times the reference value or significant rise in troponin T values), and target vessel revascularisation. Procedural complications recorded included myocardial infarction, emergency CABG, arterial complications, aortic/coronary dissection, side branch occlusion, and arrhythmia. Procedural complications were recorded at the time of the procedure and inhospital complications were entered into the database at the time of discharge. Repeated PCI rates due to target vessel revascularisation were identified from the PCI database and subsequent surgical revascularisation rates were calculated from analysis of the surgical database. All-cause mortality data was recorded as of 10 August 2011 and obtained via the National Institute of Cardiovascular Outcomes Research (NICOR) BCIS database [11]. This national database is periodically linked to the UK Office of National Statistics and provides live/death status of treated patients. Only patients who had complete database records and National Health Service unique numbers (allowing live/death status to be assessed) were included in the analysis. Major and minor bleeding were defined as per the TIMI bleeding criteria. Criteria for major bleeding were any intracranial bleeding; overt bleeding associated with a ≥5 g/dL drop in haemoglobin and/or ≥15% absolute decrease in haematocrit; or fatal bleeding resulting in a death within 7 days. Criteria for minor bleeding were overt bleeding associated with 3–5 g/dL drop in haemoglobin and/or 10–15% absolute decrease in haematocrit; occult bleeding associated with a ≥4 g/dL drop in haemoglobin concentration and/or ≥12% absolute decrease in haematocrit; or any overt bleeding which does not meet any of the previous criteria and requires medical or surgical treatment, prolonged hospital stay, or prompting unscheduled medical assessment and investigation. A retrospective data quality audit of 100 randomly selected medical records established that 94.8% of data fields, including complications, were entered correctly into the database.

### 2.1. Ethics

The data were collected as part of a national cardiac audit and all patient identifiable fields were removed prior to analysis. The Local Ethics Committee advised us that formal ethical approval was not required.

### 2.2. Statistical Analysis

Continuous data with a normal distribution are presented as mean ± standard deviation (SD) and skewed data as median ± IQR. Clinical characteristics of patients were compared using the Pearson chi-squared test for categorical variables and Student's *t*-test for continuous variables. We calculated the Kaplan-Meier product limits for cumulative probability of reaching an end point and used the log rank test for evidence of a statistically significant difference between the groups. Time was measured from the first admission for a procedure to outcome (all-cause mortality). The Cox regression analysis was used to estimate hazard ratios for the effect of GP IIb/IIIa inhibitor use in age-adjusted and fully adjusted models, based on covariates (*P* < 0.05) associated with the outcome. Included covariates in the age-adjusted model were sex, history of hypertension, history of hypercholesterolaemia, smoking status, previous myocardial infarction, previous coronary artery bypass grafting, previous PCI, previous stroke, history of peripheral vascular disease, diabetes mellitus, history of chronic kidney disease, radial approach, left anterior descending coronary artery intervention, presence of chronic total occlusion on angiography, presence of multivessel disease on angiography, drug-eluting stent use, and successful angiographic result following PCI. The proportional hazards assumption was evaluated by examining log (−log) survival curves and additionally was tested with Schoenfeld's residuals. The proportional hazard assumption was satisfied for all outcomes evaluated. A propensity score analysis was carried out using a nonparsimonious logistic regression model comparing patients split by GP IIb/IIIa use. Multiple variables were included in the model, including all variables with significant interactions.

*Propensity Score Analysis*. In order to investigate whether or not any association of GP IIb/IIIa inhibitor use on clinical outcomes was related to procedural risk, we created a propensity score to assess the baseline risk of each patient who underwent PCI. Multiple logistic regression with GP IIb/IIIa use as the dependent variable was used to develop the propensity score [12, 13]. All potential confounding variables remained in the model regardless of level of statistical significance on univariate analysis. The regression model was then used to generate the predicted probability of GP IIb/IIIa inhibitor use for each patient (i.e., their “propensity score”), which ranged between 0 and 1. We then undertook a regression adjustment incorporating the propensity score into a proportional hazard model as a covariate. We used SPSS for Windows and Mac version 20 for all analyses.

## 3. Results

Of the 3047 patients, 1294 (42.5%) were treated with GP IIb/IIIa inhibitors. Of the patients treated with GP IIb/IIIa inhibitors, the majority 1,092 patients (84.4%) received abciximab. 135 patients received tirofiban (10.4%) and 67 received eptifibatide (5.18%). Follow-up was for a median of 4.6 years (IQR range: 3.0–6.2 years).

### 3.1. Patient Characteristics (Table 1)

Patients treated with GP IIb/IIIa inhibitors were younger and more likely to be smokers. They had fewer comorbidities, being significantly less likely to have suffered a previous myocardial infarction (MI) or cerebrovascular accident (CVA), hypertension, hypercholesterolaemia, renal disease, and peripheral vascular disease (PVD). They were also less likely to have previously undergone PCI.

### 3.2. Procedural Characteristics (Table 2)

Patients treated with GP IIb/IIIa inhibitors were significantly more likely to undergo the procedure via the femoral route, receive intervention of the LAD, and have multivessel intervention. They were also more likely to undergo PCI with drug-eluting stents and utilise a pressure wire prior to the PCI. Patients receiving GP IIb/IIIa inhibitors were more likely to have a successful angiographic result after PCI than those who did not.

### 3.3. Procedural Outcomes (Table 3)

Inhospitable MACE rates were similar between those patients treated with GP IIb/IIIa inhibitors and those who were not. However, patients treated with GP IIb/IIIa inhibitors had higher rates of inhospitable Q wave MI. The major bleeding rate and total bleeding rate were significantly higher in the GP IIb/IIIa group, though the minor bleeding rate was not significantly different.

### 3.4. Long-Term Outcomes

#### 3.4.1. All-Cause Mortality (Figure 1)

**Figure 1:**
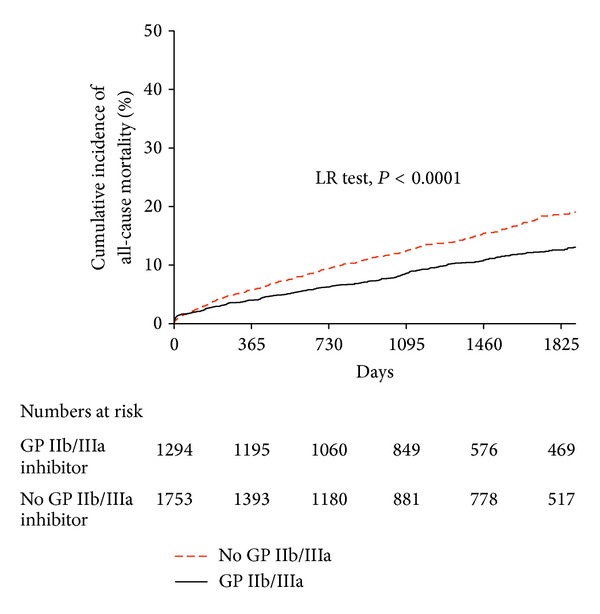
The unadjusted Kaplan-Meier curves showing cumulative incidence of all-cause mortality comparing patients treated with GP IIb/IIIa inhibitors to those not treated with them. Mortality was significantly improved amongst patients treated with GP IIb/IIIa inhibitors (*P* < 0.0001).

The unadjusted Kaplan-Meier estimates of all-cause mortality showed decreased rates of mortality for patients treated with GP IIb/IIIa inhibitors versus those who were not (*P* < 0.0001; Figure 1). Analysis of specific GP IIb/IIIa inhibitors showed decreased mortality associated with the use of abciximab (1,092 patients; *P* < 0.001) and tirofiban (135 patients; *P* = 0.003) versus no GP IIb/IIIa inhibitor use. However, eptifibatide (67 patients) showed a nonsignificant trend for decreased mortality (*P* = 0.110). There was no significant difference between agents.

#### 3.4.2. Major Adverse Cardiac Events (Figure 2)

**Figure 2:**
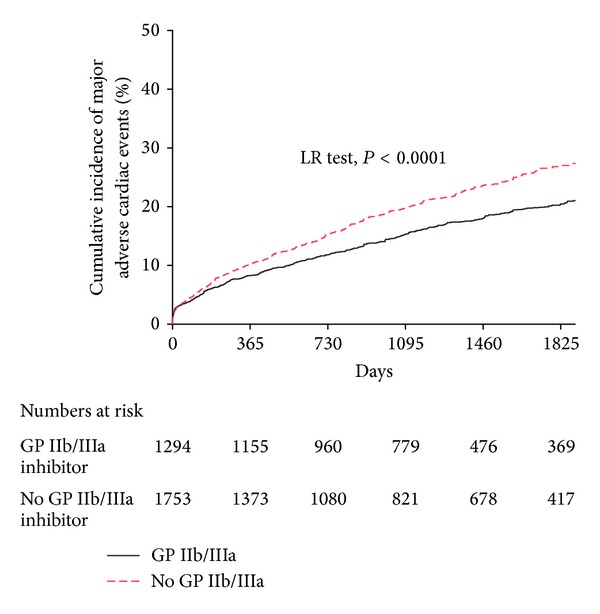
The unadjusted Kaplan-Meier curves showing cumulative incidence of long-term MACE comparing patients treated with GP IIb/IIIa inhibitors to those not treated with them. MACE were significantly improved amongst patients treated with GP IIb/IIIa inhibitors (*P* < 0.0001).

Kaplan-Meier estimates showed decreased rates of MACE (*P* < 0.0001; Figure 2) for patients treated with GP IIb/IIIa inhibitors versus those not. There was no difference between the different types of GP IIb/IIIa inhibitor.

#### 3.4.3. The Cox Regression Analysis

**Figure 3:**
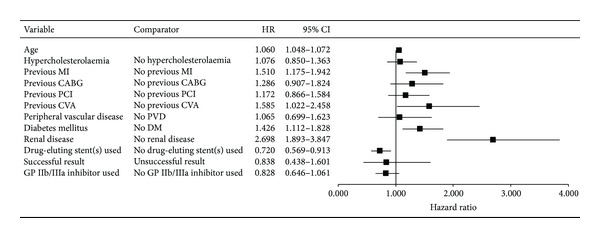
The multivariate Cox regression analysis for hazard of death (survival). Multivariate analysis failed to show a significant improvement in mortality with GP IIb/IIIa inhibitor use. In addition to increased patient age, a history of myocardial infarction (MI), cerebrovascular accident (CVA), diabetes mellitus (DM), and renal disease remained significant predictors of increased mortality. Drug-eluting stents continued to be associated with improved survival.

**Figure 4:**
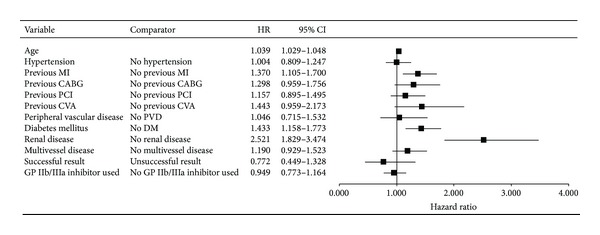
The multivariate Cox regression analysis for hazard of MACE. Multivariate analysis failed to show a significant decrease in the hazard of MACE with GP IIb/IIIa inhibitor use. In addition to increased patient age, a history of myocardial infarction (MI), diabetes mellitus (DM), and renal disease remained significant predictors of increased hazard of MACE.

The age-adjusted Cox regression analysis showed a reduction in the hazard of death (hazard ratio: 0.704; 95% confidence interval: 0.570–0.868; *P* = 0.001) and MACE (hazard ratio: 0.832; 95% confidence interval: 0.699–0.992) for patients treated with GP IIb/IIIa inhibitors. However, after multivariate adjustment the benefits in survival (hazard ratio: 0.828; 95% confidence interval: 0.646–1.061; *P* = 0.136; Figure 3) did not persist. Similarly, after multivariate analysis, GP IIb/IIIa inhibitor use was not associated with a reduction in MACE (hazard ratio: 0.949; 95% confidence interval: 0.773–1.164; *P* = 0.614; Figure 4). All covariates in this multivariate model and their hazard ratios (HRs) are shown in Figures 3 and 4. Significant variables are emboldened.

#### 3.4.4. Propensity Analysis

After correcting for propensity score, there were no significant differences in mortality or MACE rates between patients receiving and not receiving GP IIb/IIIa inhibitors. The risk ratio associated with GP IIb/IIIa use compared with GP IIb/IIIa inhibitor naive across all quintiles of baseline risk was 1.10 (95% CI: 0.86–1.41) for mortality and 1.14 (0.90–1.45) for MACE.

## 4. Discussion

This study, using data from a large PCI registry, is one of the largest observational studies yet performed specifically assessing the effect of GP IIb/IIIa receptor inhibitors on long-term outcomes in patients undergoing PCI after NSTE-ACS with concomitant routine oral P2Y_12_ inhibitor use. No significant association was seen between GP IIb/IIIa inhibitor use and either long-term mortality or long-term MACE rates after correction for confounding variables. GP IIb/IIIa inhibitor use was associated with an increased risk of major bleeding.

There is a lack of long-term follow-up data for the use of GP IIb/IIIa inhibitors in patients following PCI for NSTE-ACS with trials mainly having assessed short-term effects even prior to routine oral P2Y_12_ inhibitor blocker use. A large meta-analysis of 29,570 patients showed a small but significant reduction in death or MI at 30 days (odds ratio: 0.91; *P* = 0.02) in patients with NSTEMI/UA. This meta-analysis included 6 trials: PRISM [14], PRISM-PLUS [6], PARAGON A [15], PARAGON B [16], PURSUIT [5], and GUSTO IV [17]. Of these, only PARAGON B showed a significant improvement on independent analysis at 30 days (odds ratio 0.58; *P* = 0.04), and there was no significant improvement in mortality. Clopidogrel, prasugrel, and ticagrelor act upstream of GP IIb/IIIa mediated platelet aggregation and all patients in our study received a loading dose of 300 or 600 mg clopidogrel. Our patients may therefore have benefitted less from the additional antiplatelet effect of GP IIb/IIIa inhibitors.

More recently, ISAR-REACT 2 [7] included 2,022 “high risk” NSTE-ACS patients treated with 500 mg aspirin and 600 mg clopidogrel prior to abciximab treatment. A subgroup analysis of NSTEMI patients was performed. The composite end point of death MI or urgent revascularisation was reduced in NSTEMI patients (RR: 0.71; 95% CI: 0.54–0.95; *P* = 0.02; *P* = 0.07 for interaction). However, mortality was not significantly reduced (RR: 0.69, 95% CI: 0.32–1.47, *P* = 0.33) and by one-year follow-up this nonsignificant trend had almost disappeared (RR: 0.91, 95% CI: 0.61–1.37, *P* = 0.66) [8]. Interestingly, in contrast to our data, this study showed no increased risk of major bleeding with these agents (RR: 1.00, 95% CI: 0.50–2.08).

GP IIb/IIIa inhibitors, through potent inhibition of platelet aggregation, are thought to reduce periprocedural thrombosis. This could be expected to reduce early recurrent myocardial infarction, though any reductions in longer-term mortality through such a mechanism may be much smaller, explaining why no trials or meta-analyses have shown a survival benefit from these agents. Furthermore, any mortality benefit gained through reducing MI may be offset by complications such as bleeding; indeed, our study showed a significantly increased risk of both major bleeding and total bleeding in those treated with GP IIb/IIIa inhibitors. This finding is supported by evidence from registries indicating that adverse events from these agents may be more common than indicated by trials [10]. However, there was a higher rate of femoral access amongst the GP IIb/IIIa cohort, potentially leading to increased bleeding risk.

In our study, patients receiving GP IIb/IIIa inhibitors were younger and had fewer comorbidities suggesting underuse of GP IIb/IIIa inhibitors in higher risk patients. Whilst patients of varying age and comorbidity may be expected to benefit equally from the potent-antiplatelet effects of these agents during PCI for NSTEMI, there is evidence that increasing age is an independent risk factor for bleeding following GP IIb/IIIa inhibitor use [18]. Furthermore, patients with comorbidities are likely to be less tolerant of haemorrhage than younger, fitter patients. Indeed, one explanation for the observation of higher postmarketing complications from these agents has been the increased use of GP IIb/IIIa inhibitors in elderly patients; the average age of patients whose deaths are directly attributable to GP IIb/IIIa inhibitors in postmarketing surveillance is 69 years, compared with 60 years across all patients in various randomised trials [10].

It is possible that our results are influenced by our choice of GP IIb/IIIa agents. However, our data fail to show superiority for any single agent. Furthermore, the TARGET trial showed abciximab to be superior to tirofiban at 30 days [19]. Whilst higher doses of tirofiban may be more effective [20, 21], the vast majority (84.4%) of our patients who received GP IIb/IIIa inhibitor were administered abciximab. We therefore feel it is unlikely that a possible benefit from GP IIb/IIIa inhibitors has been obscured through the use of less effective agents.

## 5. Study Limitations

Our study is a consecutive but retrospective observational analysis from a single center's experience. As this was an observational study the findings may have been subject to confounding factors that we have been unable to control for. This may include adherence to evidence-based therapies after discharge, which is known to be associated with poor outcomes. However, our dataset includes most major clinical variables known to affect outcome, which would support the validity of our results.

The inhospital mortality rates in this study are significantly lower (0.54% and 0.74% for those receiving and not receiving GP IIb/IIIa inhibitors, resp.) than previously reported in NSTEMI patients [22]. This may be at least partially explained by the selective nature of our high-volume cardiac centre. As our centre lacks an emergency department, the cohort of patients it receives may differ from those seen in general hospitals. It is therefore possible that our results may not fully reflect the benefits and risks of GP IIb/IIIa inhibitor use in a population of NSTEMI patients typically encountered in other hospitals.

We were able to analyze only 92% of individuals who had PCI during the study period due to incomplete datasets. We cannot account for the effects of residual confounding or of selection bias caused by exclusion of this 8% of patients. However, this is unlikely as the distribution of GP IIb/IIIa inhibitor use was the same in the excluded and analyzed groups (data not shown). Finally although our multivariate analysis revealed no improvement in survival for patients treated with GP IIb/IIIa use, it is possible that we had insufficient power to detect a difference in mortality.

## 6. Conclusion

Although GP IIb/IIIa inhibitor use appeared to be associated with improved outcome after PCI for NSTEMI, patients who received GP IIb/IIIa inhibitors had a different risk profile to patients who did not receive GP IIb/IIIa inhibitors. Therefore after multivariate adjustment including the use of a propensity score, we observed no improvement in MACE or survival in those patients who received GP IIb/IIIa inhibitors. GP IIb/IIIa inhibitor use was associated with an increased risk of major and total bleeding.

## Figures and Tables

**Table 1:** Baseline characteristics.

	No GP IIb/IIIa inhibitor used (*n* = 1753)	GP IIb/IIIa inhibitor used (*n* = 1294)	Significance (*P*)
Age	65.1 (±12.4)	62.0 (±12.2)	<0.001*
Gender (female)	501 (28.6%)	336 (26.0%)	0.119
Previous MI	577 (32.9%)	352 (27.2%)	0.001*
Previous CABG	160 (9.12%)	94 (7.26%)	0.073
Previous PCI	336 (19.2%)	154 (11.9%)	<0.001*
Current smoker	417 (23.8%)	430 (33.2%)	<0.001*
Hypertension	1079 (61.6%)	700 (54.1%)	<0.001*
Hypercholesterolaemia	1044 (59.6%)	617 (47.4%)	<0.001*
DM	448 (25.6%)	315 (24.3%)	0.447
Renal disease	103 (5.88%)	36 (2.78%)	<0.001*
Previous CVA	63 (3.59%)	26 (2.01%)	0.012*
PVD	82 (4.68%)	38 (2.94%)	0.014*
Cardiogenic shock	20 (1.14%)	21 (1.62%)	0.269

*P value < 0.05.

**Table 2:** Procedural characteristics.

	No GP IIb/IIIa inhibitor used (*n* = 1753)	GP IIb/IIIa inhibitor used (*n* = 1294)	Significance (*P*)
Access route			
Radial	691 (39.4%)	383 (29.6%)	<0.001*
Brachial	1 (0.06%)	2 (0.15%)	0.267
Femoral	1061 (60.5%)	909 (70.2%)	<0.001*
Target vessel(s)			
Right coronary artery	633 (36.1%)	499 (38.6%)	0.172
Left main coronary artery	36 (2.05%)	38 (2.93%)	0.123
Left anterior descending artery	662 (37.8%)	690 (53.3%)	<0.001*
Left circumflex artery	496 (28.3%)	386 (38.3%)	0.374
Bypass graft	94 (5.36%)	68 (5.26%)	0.935
Multivessel disease	401 (22.9%)	312 (24.1%)	0.436
Multivessel intervention	347 (19.8%)	401 (31.0%)	<0.001*
Drug-eluting stent used	909 (51.9%)	757 (58.5%)	<0.001*
IVUS used	86 (4.91%)	56 (4.23%)	0.487
Pressure wire used	88 (5.02%)	14 (1.08%)	<0.001*
Success	1697 (96.8%)	1286 (99.4%)	<0.001*

*P value < 0.05.

**Table 3:** Procedural outcomes.

	No GP IIb/IIIa inhibitor used (*n* = 1753)	GP IIb/IIIa inhibitor used (*n* = 1294)	Significance (*P*)
Inhospital			
MACE	24 (1.37%)	22 (1.70%)	0.457
Death	13 (0.74%)	7 (0.54%)	0.651
Q wave MI	5 (0.29%)	12 (0.93%)	0.025*
Reintervention PCI	5 (0.29%)	6 (0.46%)	0.543
Emergency CABG	3 (0.17%)	1 (0.08%)	0.641
Coronary dissection/perforation	25 (1.43%)	26 (2.01%)	0.253
CVA	2 (0.11%)	0 (0.00%)	0.511
Side branch occlusion	10 (0.57%)	9 (0.70%)	0.817
Major bleeding	6 (0.34%)	14 (1.08%)	0.021*
Minor bleeding	36 (2.05%)	40 (3.09%)	0.078
Total bleeding	42 (2.40%)	54 (4.17%)	0.0063*
Heart block requiring pacing	2 (0.11%)	1 (0.08%)	0.578
DC cardioversion	4 (0.23%)	2 (0.15%)	1.00

*P value < 0.05.
